# World Kidney Day 2014: increasing awareness of chronic kidney disease and aging

**DOI:** 10.12861/jrip.2014.02

**Published:** 2013-11-19

**Authors:** German T Hernandez, Hamid Nasri

**Affiliations:** ^1^Department of Internal Medicine, Division of Nephrology & Hypertension, Paul L. Foster School of Medicine, Texas Tech University Health Sciences Center at El Paso, El Paso, Texas, U.S.A.; ^2^Department of Nephrology, Division of Nephropathology, Isfahan University of Medical Sciences, Isfahan, Iran

**Keywords:** Chronic kidney disease, Acute kidney injury, Hypertension, World kidney day

**Implication for health policy/practice/research/medical education:**

In 2014 World Kidney Day (WKD) will focus on chronic kidney disease and aging. The mission of WKD is to raise awareness so that everyone cares for their kidneys and, if appropriate, check to assess if they are at risk for kidney disease. Prevention of kidney disease, early detection, and subsequent kidney protection are critical aims for WKD.

Last year, the International Society of Nephrology (ISN) and the International Federation of Kidney Foundations (IFKF), focused the World Kidney Day (WKD) of 2013 on acute kidney injury (1). In 2014 the WKD, however will focus on increasing awareness of chronic kidney disease and aging. WKD is a joint initiative of ISN and IFKF. WKD is a global health awareness campaign focusing on the importance of renal disease and reducing the frequency and impact of kidney disease and its associated health problems worldwide (1). The campaign is celebrated each year on the second Thursday of March in many countries (1). The term acute kidney injury was suggested to reflect the wide spectrum of traditional acute kidney failure. Acute kidney injury is particularly common in hospitalized individuals and its presence confers an increased risk of subsequent chronic kidney disease, increased hospital length of stay, and a higher risk of death (1,2). However, the impact of acute renal injury on the progression of chronic kidney disease is not well understood. Indeed, acute kidney injury can cause end-stage kidney failure directly, as well as increase the risk of developing incident chronic kidney disease or worsening progression of underlying chronic kidney disease (1-3). Furthermore, the duration, severity and frequency of acute kidney injury appear to be important predictors of poor patient outcomes. In addition, chronic kidney disease is an important risk factor for the development of acute kidney injury (2-4). Various studies provide the clinical findings and the bidirectional nature of the association between acute kidney injury and chronic kidney disease. Chronic kidney disease is a long-term condition and defined as the slow loss of renal function over time (1-3). Over the last decade, interest in the risk factors, incidence, rate of progression, and clinical outcomes of chronic kidney disease has been increasing as a result of high prevalence and increasing awareness of CKD. In the early stages of the disease process, patients with chronic kidney disease frequently experience no symptoms at all (2-5). However, even in the absence of symptoms, chronic kidney disease negatively affects various organs, raises the risk of cardiovascular events, can progress to end-stage kidney failure, and increases the risk of hospitalization and death (6,7). Worldwide there is an increasing prevalence of end-stage kidney disease (2-5). The rise in prevalence of end-stage kidney disease continues in spite of at least two decades of intensified kidney-protection modalities, consisting of optimal hypertension control, acceptable glycemic control in diabetics, smoking cessation, and the wide use of renin-angiotensin-aldosterone system (RAAS) blockade in both diabetic and non-diabetic CKD (1-5). The current thinking is that chronic kidney disease progresses to end-stage kidney failure or death at an increasing rate. Indeed, except for early premature cardiovascular death, progression from kidney parenchymal injury to end-stage kidney disease is the final common pathway for chronic nephropathies, which seems to be largely independent of the initial renal insult. The most important event is increased glomerular capillary pressure, which impairs glomerular impermeability to proteins and permits an excessive amount of protein to reach the proximal tubules (1-4). The secondary event, tubular reabsorption of filtered protein, contributes to kidney interstitial damage by activating intracellular mechanisms, consisting of up-regulation of the genes encoding vasoactive and inflammatory mediators (2,4). However, interstitial inflammation and progression of disease can be controlled with angiotensin-converting enzyme inhibition or angiotensin receptor blockade, which intensify the glomerular impermeability barrier to proteins and thus limit proteinuria and the filtered protein-dependent inflammatory signals. Various clinical findings strongly suggest that remission can now be achieved in some patients with chronic kidney disease (3,5). Diabetes and hypertension are the leading causes of chronic kidney disease and their incidence is increasing at an alarming rate (1,2,6). The most important mission of WKD 2014 in regards to chronic kidney disease and aging is slowing the progression of chronic kidney disease in elderly patients (2-4). It should be noted that, chronic kidney disease requires careful additional management, not only to diminish the risk of progression to end-stage kidney disease but also because it is probably one of the greatest risk factors for cardiovascular mortality and morbidity (4-6). However, the rate of progression of chronic kidney disease shows considerable inter-individual variability and is affected by various factors (2-6). Although previous investigations have shown a wide range of risk factors for chronic kidney disease, the real specific role of each factor is not completely clear. Focusing on risk factors such as, age, sex, high blood pressure, diabetes mellitus, ethnicity, metabolic syndrome, family history of chronic kidney disease and proteinuria may help in the early identification of patients with CKD and slow their progression through appropriate interventions (2-4). Recently however, attention has also been focused on other novel factors, such as fibroblast growth factor 23 (FGF 23), asymmetric dimethylarginine (ADMA), calcium–phosphate metabolism and adiponectin (1-6). The ability to identify these predictors or aggravators would enable us to target high-risk individuals and offer them intensive medical surveillance and management, to reduce the rate of progression to end-stage kidney failure, cardiovascular events, or death (5-8). The mission of WKD is to raise awareness so that everyone cares for their kidneys and, if appropriate, check to assess if they are at risk for kidney disease. Prevention of kidney disease, early detection, and subsequent kidney protection are critical aims for WKD.

## Authors’ contributions

GTH and HN contributed as authors of the manuscript.

## Conflict of interests

The author declared no competing interests.

## Ethical considerations

Ethical issues (including plagiarism, data fabrication, double publication) have been completely observed by the author.

## Funding/Support

None.
